# Dataset on the spatial distribution of groundwater quality for pH, Electrical Conductivity (EC), Total Hardness (TH), Ca^2+^, Mg^2+^, HCO_3_^−,^ F^−^, and NO_3_^−^ in Dodoma, Singida, and Tabora regions located in central Tanzania

**DOI:** 10.1016/j.dib.2023.109239

**Published:** 2023-05-16

**Authors:** Jonas Didas Chondo, Andrew Toyi Banyikwa

**Affiliations:** University of Dodoma, College of Natural and Mathematical Sciences (CNMS), Department of Chemistry, Dodoma, P.O.BOX 338, United Republic of Tanzania

**Keywords:** Water quality, Pollution, Dodoma, Singida, Tabora, Central Tanzania

## Abstract

Groundwater is an important source of water for drinking and irrigation purposes in semi-arid regions like central Tanzania. Groundwater quality is degraded by anthropogenic and geogenic pollution. Anthropogenic pollution depends on the disposal of contaminants from human activities into the environment, which can leach and pollute groundwater. Geogenic pollution depends on the presence and dissolution of mineral rocks. High geogenic pollution is observed in aquifers that are rich in carbonates, feldspars, and mineral rocks. Consumption of polluted groundwater has negative health effects. Therefore, protection of public health necessitates the evaluation of groundwater in order to identify a general pattern and spatial distribution of groundwater pollution. A literature search uncovered no publications that describe the spatial distribution of hydrochemical parameters across central Tanzania. Central Tanzania is located within East Africa Rift Valley, Tanzania craton and is made up of Dodoma, Singida and Tabora regions. To fill the gap, this article contains a data set for pH, Electrical Conductivity (EC), Total Hardness (TH), Ca^2+^, Mg^2+^, HCO_3_^−^, F^−^, and NO_3_^−^ from 64 groundwater samples collected from Dodoma region (22 samples), Singida region (22 samples) and Tabora region (20 samples). Data collection covered a total distance of 1344 km, which was divided into east-west along B129, B6, and B143 roads and north-south along A104, B141, and B6 roads. The present dataset can be used to model the geochemistry and spatial variation of physiochemical parameters across these three regions.

Specifications table

**Table:** 

Subject	Environmental Science				

Specific subject area	Environmental Chemistry, Pollution				
Type of data	Table Figures				
How the data were acquired	pH and EC measurements were done using a pH metre from Hanna Instruments (HI98130). The phenol disulfonic method and Cary 60 Uv-Vis spectrophotometer from Agilent Technologies were used to determine the concentration of NO_3_^−^. Determination of F^−^ was done using a The SPADNS method and Hach DR900 Handheld Colorimeter (DR/890 Colorimeter). TH and HCO_3_^−^ were determined using titration methods. TH as CaCO_3_ was determined using EDTA and Eriochrome Black T (EBT) indicator. HCO_3_^−^ was determined using 0.05 M of H_2_SO_4_ and POP (Phenolphthalein) followed by MO (Methyl orange) indicators.				
Data format	Raw				
Description of data collection	A total of 64 water samples were collected in all. 22 samples from Dodoma and Singida and 20 samples from Tabora. All samples were collected from boreholes that are publicly accessible. The data collection covered a total distance of 1344 km. GPS coordinates of sample collection sites were recorded using a Garmin Nuvi 255 w GPS receiver. All samples were collected within a three-day period in the month of August of 2020. pH, temperature, and EC were measured on site. The levels of TH, HCO_3_^−^, NO_3_^−^, and F^−^ were determined within 24 h of sample collection. The samples were kept in a 1 L polythene plastic bottle washed with distilled and sample water. Samples were stored in the cold room (4 °C) before analysis.				

Data source location	GPS coordinates, region, and the name of the location where the samples were collected				
	
	S/N		Coordinates	Coordinates	Road
	
	1	1	S04°32.655′	E035°41.881′	Dodoma-North-South
	2	2	S04°44.202′	E035°49.650′	Dodoma-North-South
	3	3	S04°55.119′	E035°48.352′	Dodoma-North-South
	4	4	S05°14.010′	E035°53.489′	Dodoma-North-South
	5	5	S05°34.223′	E035°50.674′	Dodoma-North-South
	6	6	S05°47.558′	Eo35°48.097′	Dodoma-North-South
	7	7	S05°50.787′	Eo35°45.718′	Dodoma-North-South
	8	8	S06°01.564′	E035°45.282′	Dodoma-North-South
	9	9	S06°10.170′	E035°47.213′	Dodoma-North-South
	10	10	S06°08.173′	E036°02.433′	Dodoma-North-South
	11	11	S06°29.893′	E035°50.271′	Dodoma-North-South
	12	12	S06°36.458′	E035°55.817′	Dodoma-North-South
	13	13	S06°51.702′	E036°02.402′	Dodoma-North-South
	14	14	S07°08.411′	E035°53.728′	Dodoma-North-South
	15	15	S06°08.272′	E036°51.053′	Dodoma-East-West
	16	16	S06°04.959′	E036°34.814′	Dodoma-East-West
	17	17	S06°06.232′	E036°19.257′	Dodoma-East-West
	18	18	S06°08.173′	E036°02.433′	Dodoma-East-West
	19	19	S06°10.170′	E035°47.213′	Dodoma-East-West
	20	20	S06°05.355′	E035°36.843′	Dodoma-East-West
	21	21	S05°59 .57′'	E035°22.59′	Dodoma-East-West
	22	22	S05°57.09′	E035°18.59′	Dodoma-East-West
	23	1	S04°21.562′	E034°12.069′	Singida-North South
	24	2	S04°24.486′	E034°23.869′	Singida-North South
	25	3	S04°25.637′	E034°28. 392′	Singida-North South
	26	4	S05°04.518′	E032°41.659′	Singida-North South
	27	5	S04°36.094′	E034°35.273′	Singida-North South
	28	6	S04°38.536′	E034°39.666′	Singida-North South
	29	7	S04°41.240	E035°060.311	Singida-North South
	30	8	S.04°42.662′	E035°04.088′	Singida-North South
	31	9	S.04°48.570′	E034°55.752′	Singida-North South
	32	10	S.04°49.890′	E043°454.181′	Singida-North South
	33	11	S.04°49.755′	E034°49.110′	Singida-North South
	34	12	S.04°49.549′	E034°46.574′	Singida-North South
	35	13	S04°49.940′	E034°45.134′	Singida-North South
	36	14	S05°06.762′	E034°46.022′	Singida-North South
	37	15	S05°20.476′	E034°46.360′	Singida-North South
	38	16	S05°43.327′	E034°41.888′	Singida-North South
	39	17	S05°52.784′	E035°07.512′	Singida-East-West
	40	18	S05°49.731′	E034°58.018′	Singida-East-West
	41	19	S05°45.334′	E034°50,496′	Singida-East-West
	42	20	S05°43.327′	E034°41.888′	Singida-East-West
	43	21	S05°41.804′	E034°29.642′	Singida-East-West
	44	22	S05°35.480′	E034°14.041′	Singida-East-West
	45	1	S04°12.751′	E033°11.125′	Tabora-North-South
	46	2	S04°43.482′	E033°09.876′	Tabora-North-South
	47	3	S04°57.706′	E033°00.406′	Tabora-North-South
	48	4	S05°02. 858′	E032°48. 018′	Tabora-North-South
	49	5	S05°04.110′	E032°47.117′	Tabora-North-South
	50	6	S05°14.717′	E032°43.412′	Tabora-North-South
	51	7	S05°19. 024′	E032°41.974′	Tabora-North-South
	52	8	S05°28. 110′	E932°41.626′	Tabora-North-South
	53	9	S05°33.092′	E032°42.332′	Tabora-North-South
	54	10	S05°41.276′	E032°45 094′	Tabora-North-South
	55	11	S05°47.490′	E032°43 922′	Tabora-North-South
	56	12	S05°29.535′	E033°50.555′	Tabora-East-West
	57	13	S05°07.602′	E033°07.772′	Tabora-East-West
	58	14	S05°04.184′	E032°55.813′	Tabora-East-West
	59	15	S05°04.184′	E032°55.813′	Tabora-East-West
	60	16	S05°04.110′	E032°47.117′	Tabora-East-West
	61	17	S05°04.518′	E032°41.659′	Tabora-East-West
	62	18	S05°07.272′	E032°31.373′	Tabora-East-West
	63	19	S05°05.298′	E032°19.022′	Tabora-East-West
	64	20	S05°04.313′	E032°04.995′	Tabora-East-West

Data accessibility	Repository name: Mendeley data Data identification number: 10.17632/4j8npwgvmv.2 Direct link to data: https://data.mendeley.com/datasets/4j8npwgvmv/2[1]				

## Value of the Data

• The data set provided allows the use of pH, Electrical Conductivity (EC), Total Hardness (TH), Ca^2+^, Mg^2+^, HCO_3_^−^to elucidate the spatial distribution and dissolution of carbonate rocks within each region and across central Tanzania.
• The dataset can be reused to model the regulation of F^−^ from TH, HCO_3_^−^, and pH within each region and across central Tanzania.
• The dataset can be used as a tool to model the geochemistry of the underlying groundwater aquifer in central Tanzania.
• The dataset can be used as a tool to NO_3_^−^ pollution in central Tanzania

## 1 Objective

A literature search shows that in central Tanzania the Dodoma region is the most studied, followed by Singida, and very little data was found for the Tabora region. Dodoma and Singida regions were reported to have elevated levels of Ca^2+^,Mg^2+^, Na^+^, HCO_3_^−^, Cl^−^,SO_4_^2−^, NO_3_^−^and F ^−^ [2], [3], [4]. F ^−^ levels were found to be lower than levels reported in Arusha and Manyara regions located northeast of central Tanzania [5]. The sources of NO_3_^−^ were reported to be anthropogenic [6], [7], [8], [9], [10]. In the Dodoma region, mineralization was found to exist in the gradient and concentrated to the south-southeast [3]. No data were found that assessed the spatial distribution of physiochemical parameters across Dodoma, Singida, and Tabora regions from the north to the south and east to the west. Lack of data makes it impossible to elucidate the spatial distribution of mineralization and identify which areas contain the highest and lowest levels of groundwater pollution across central Tanzania. The datasets presented in this work were covers all three regions and were collected along the B129, B6, and B143 roads and the A104, B141, and B6 roads, which cross these three regions from east to west and from north to south, respectively. The datasets generated were for pH, EC, TH, Ca^2+^, Mg^2+^, HCO_3_^−,^ F ^−^, and NO_3_^−^.

## 2 Data Description

The datasheet that contains the raw data presented in this work is deposited in the Mendeley Data repository under the file name “Raw data submitted” [1]. The first sheet named “Calibration Curves” shows the calibration curves that were used for the levels of electrical conductivity, NO_3_^−^ and F ^−^. The second sheet, named “physiochemical parameters,” shows the GPS coordinates, the names of the roads where the samples were collected, the names of the area where the samples were collected, and the values of their physiochemical parameters. The third sheet, named “Pearson correlation,” shows Pearson correlation tables from data obtained in Dodoma, Singida, and Tabora regions. This sheet can also be used to see the rows, columns, and formulas (=PEARSON(Array1, Array2)) that were used to determine the correlations.

The second sheet with name Physiochemical parameters are presented in the Excel sheet named physiochemical parameter. Pearson correlation tables and figures are presented in the Excel sheet named Pearson correlation and figures respectively. The fourth sheet, named “Figures,” shows the presentation of raw data in figure form. This sheet shows how the figures were constructed from the raw data, also shown in the same sheet.

**Fig. 1:**
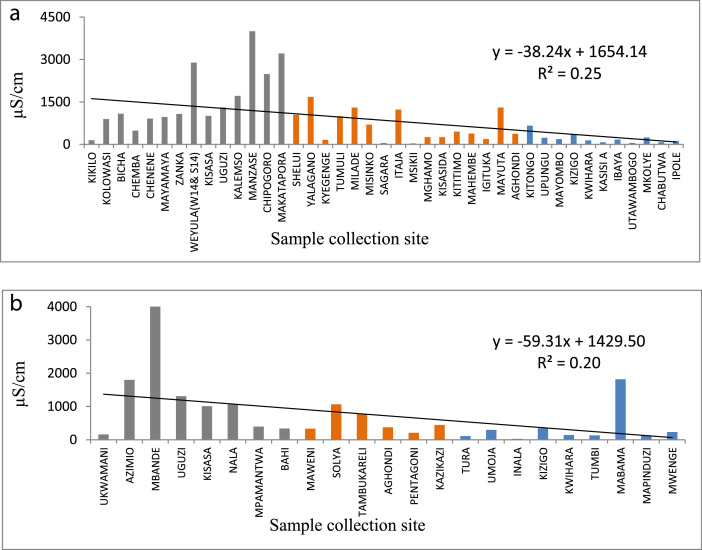
(a) EC from North to South in Dodoma region (grey), Singida region (orange) and Tabora region (blue). (b) EC across from east to west in Dodoma region (grey), Singida region (orange) and Tabora region (blue).

**Fig. 2:**
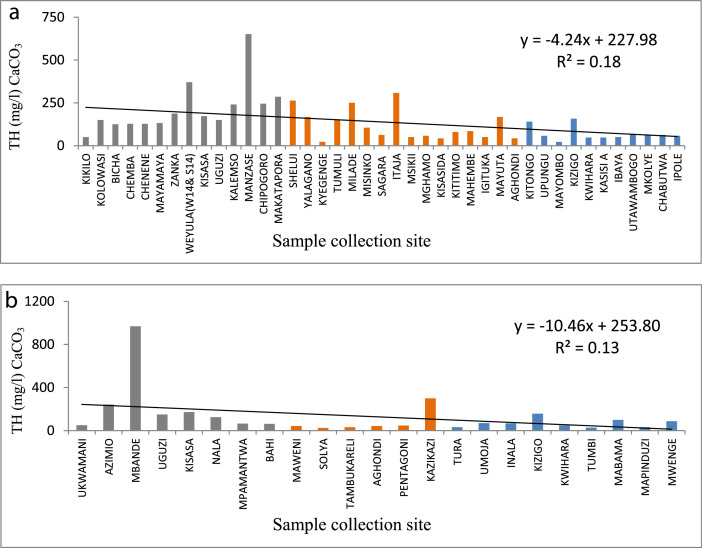
(a) TH from North to South in Dodoma region (grey), Singida region (orange) and Tabora region (blue). (b) TH from east to west in Dodoma region (grey), Singida region (orange) and Tabora region (blue).

**Fig. 3:**
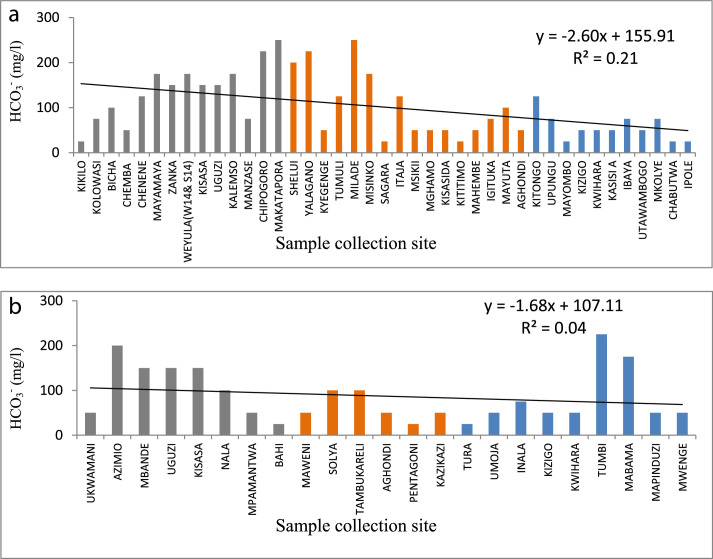
(a) HCO_3_^−^ variation from North to South in Dodoma region (grey), Singida region (orange) and Tabora region (blue). (b) HCO_3_^−^ from east to west in Dodoma region (grey), Singida region (orange) and Tabora region (blue).

**Fig. 4:**
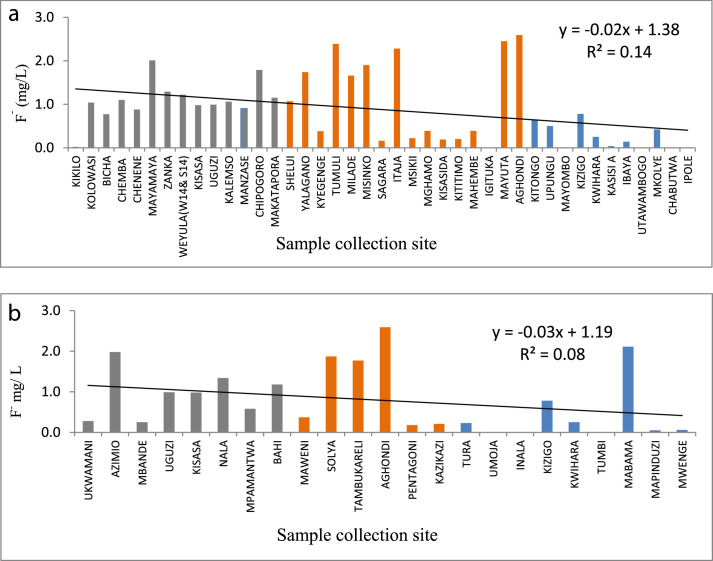
(a) F^−^ variation from North to South in Dodoma region (grey), Singida region (orange) and Tabora region (blue). (b) F^−^ variation from east to west in Dodoma region (grey), Singida region (orange) and Tabora region (blue).

**Fig. 5:**
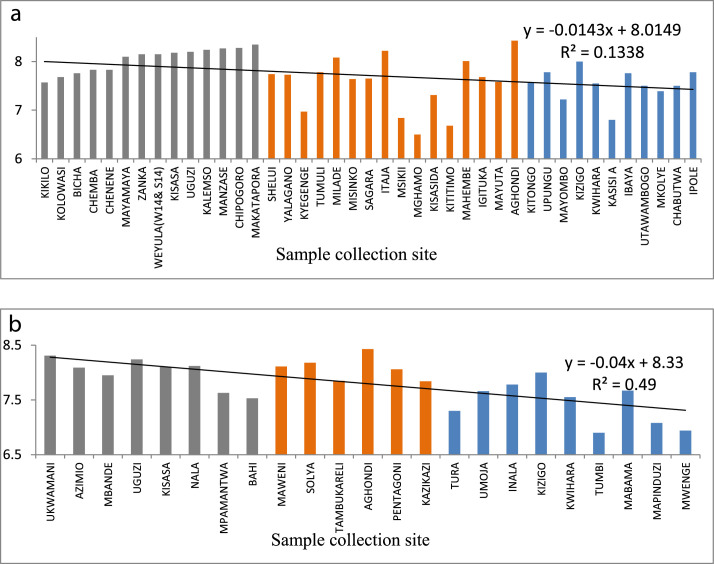
(a) pH variation from North to South in Dodoma region (grey), Singida region (orange) and Tabora region (blue). (b) pH variation from east to west North in Dodoma region (grey), Singida region (orange) and Tabora region (blue).

**Fig. 6:**
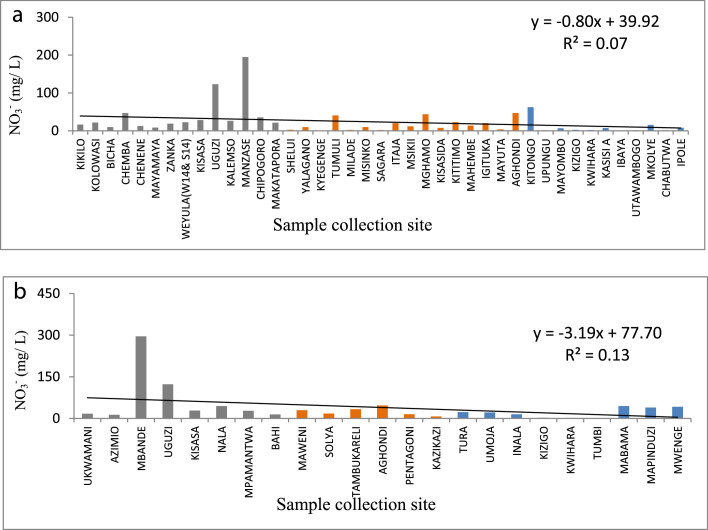
(a) NO_3_^−^variation from North to South in Dodoma region (grey), Singida region (orange) and Tabora region (blue). (b) NO_3_^−^variation from east to west in Dodoma region (grey), Singida region (orange) and Tabora region (blue).

The statistical summary, which shows mean, standard deviation, maximum, minimum, WHO guidelines, and the percent of samples that are above the WHO guideline, is presented in Table 1. Fig. 1a and b show the distribution of EC. Fig. 2a and b show the distribution of TH. Fig. 3a and b show the distribution of HCO_3_^−^. Fig. 4a and b show the distribution of F ^−^. Fig. 5a and b show the distribution of pH. Fig. 6a and b show the distribution of NO_3_^−^. Table 2, Table 3, Table 4 shows Peterson correlation tables from Dodoma, Singida, and Tabora regions respectively.

**Table 1:** Statistical summary of physicochemical parameters in Dodoma, Singida and Tabora regions.

	Maximum	Minimum	Mean and standard deviation	WHO	% Samples above WHO guideline
pH					

Dodoma	8.35	7.53	8.03± 0.25	6.5–8.5	0.00
Singida	8.43	6.5	7.66±0.52		0.00
Tabora	8.06	6.8	7.48±0.38		0.00

EC (µS/cm)					

Dodoma	4012	145	1344.61±1190.72	<2500	18.00
Singida	1678	36.1	611.23±500.79		0.00
Tabora	1818	27	294.27±383.65		0.00

TH (mg/L) CaCO_3_					

Dodoma	968.3	47.5	215.3 ± 213.92	<500	9.10
Singida	307	22.52	112±94.51		0.00
Tabora	157.6	22.62	69.61±34.63		

HCO_3_^−^ (mg/L)					

Dodoma	250	25	115.22+66.03	20–200	9.10
Singida	250	25	92.86±67.61		0.00
Tabora	225	25	79.55±53.11		0.00

NO_3_^–^ (mg/L)					

Dodoma	295.8	8.3	46.69±68.69	45	22.70
Singida	47	0.7	17.15±14.29		0.00
Tabora	62.3	1	15.1 ± 17.21		5.0

F^–^ (mg/L)					

Dodoma	2.01	0.02	0.94±0.55	0.5–1.5	13.6
Singida	2.59	0.01	1.12±0.93		45.45
Tabora	2.11	0.01	0.38±0.51		5.0

**Table 2:** Pearson correlation in Dodoma region.

	pH	NO3-	F-	TH	Ca2+	Mg2+	HCO3-	EC	TDS (PPM)
pH	1.00								
NO3-	−0.13	1.00							
F-	0.08	−0.32	1.00						
TH	−0.19	0.84	−0.13	1.00					
Ca2+	−0.22	0.84	−0.17	0.96	1.00				
Mg2+	0.80	0.80	−0.11	0.99	0.90	1.00			
HCO3-	0.43	0.02	0.57	0.28	0.20	0.31	1.00		
EC	−0.08	0.64	0.11	0.88	0.81	0.89	0.55	1.00	
TDS (PPM)	−0.06	0.70	0.06	0.93	0.86	0.93	0.52	0.99	1.00

**Table 3:** Pearson correlation in Singida region.

	pH	NO3-	F-	TH	Ca2+	Mg2+	HCO3-	EC	TDS (PPM)
pH	1.00								
NO3-	0.18	1.00							
F-	0.54	0.36	1.00						
TH	0.21	−0.35	0.24	1.00					
Ca2+	0.18	−0.06	0.50	0.66	1.00				
Mg2+	0.14	−0.41	−0.05	0.83	0.13	1.00			
HCO3-	0.23	−0.29	0.48	0.61	0.46	0.43	1.00		
EC	0.32	−0.16	0.66	0.66	0.69	0.30	0.83	1.00	
TDS (PPM)	0.31	−0.16	0.62	0.65	0.77	0.29	0.75	0.98	1.00

**Table 4:** Pearson correlation in Tabora region.

	pH	NO3-	F-	TH	Ca2+	Mg2+	HCO3-	EC	TDS (PPM)
pH	1.00								
NO3-	−0.21	1.00							
F-	0.39	0.36	1.00						
TH	0.56	0.26	0.56	1.00					
Ca2+	0.52	0.32	0.82	0.87	1.00				
Mg2+	0.50	0.18	0.28	0.94	0.63	1.00			
HCO3-	−0.15	0.26	0.44	0.08	0.30	−0.09	1.00		
EC	0.22	0.54	0.93	0.42	0.72	0.15	0.53	1.00	
TDS (PPM)	0.23	0.30	0.89	0.30	0.58	0.05	0.43	0.93	1.00

## 3 Experimental Design, Materials and Methods

### 3.1 Study Area Description

**Fig. 7:**
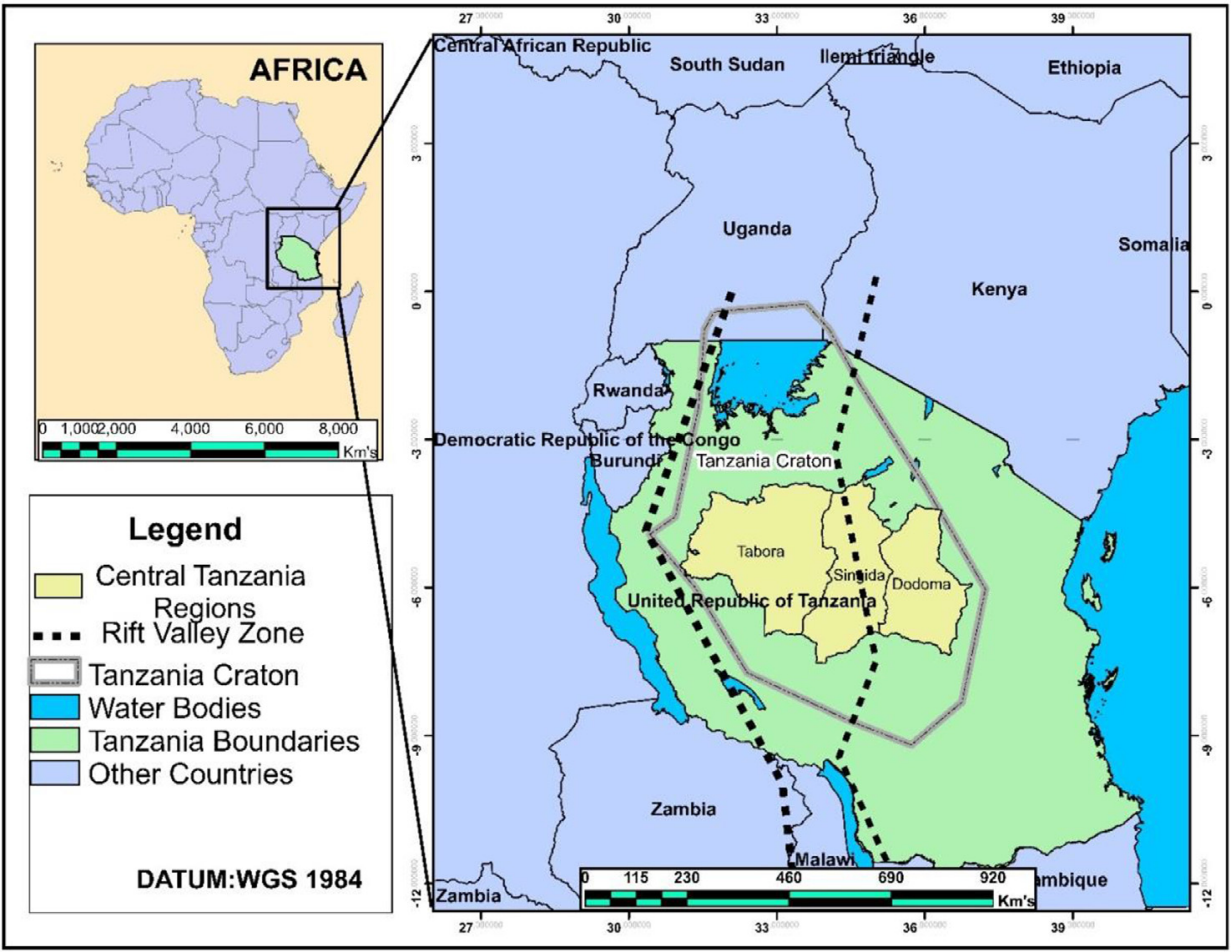
Map of Tanzania, which shows the Dodoma, Singida, and Tabora regions, the Tanzania craton, and the Rift Valley zone. This map has been modified from the published map [15].

**Fig. 8:**
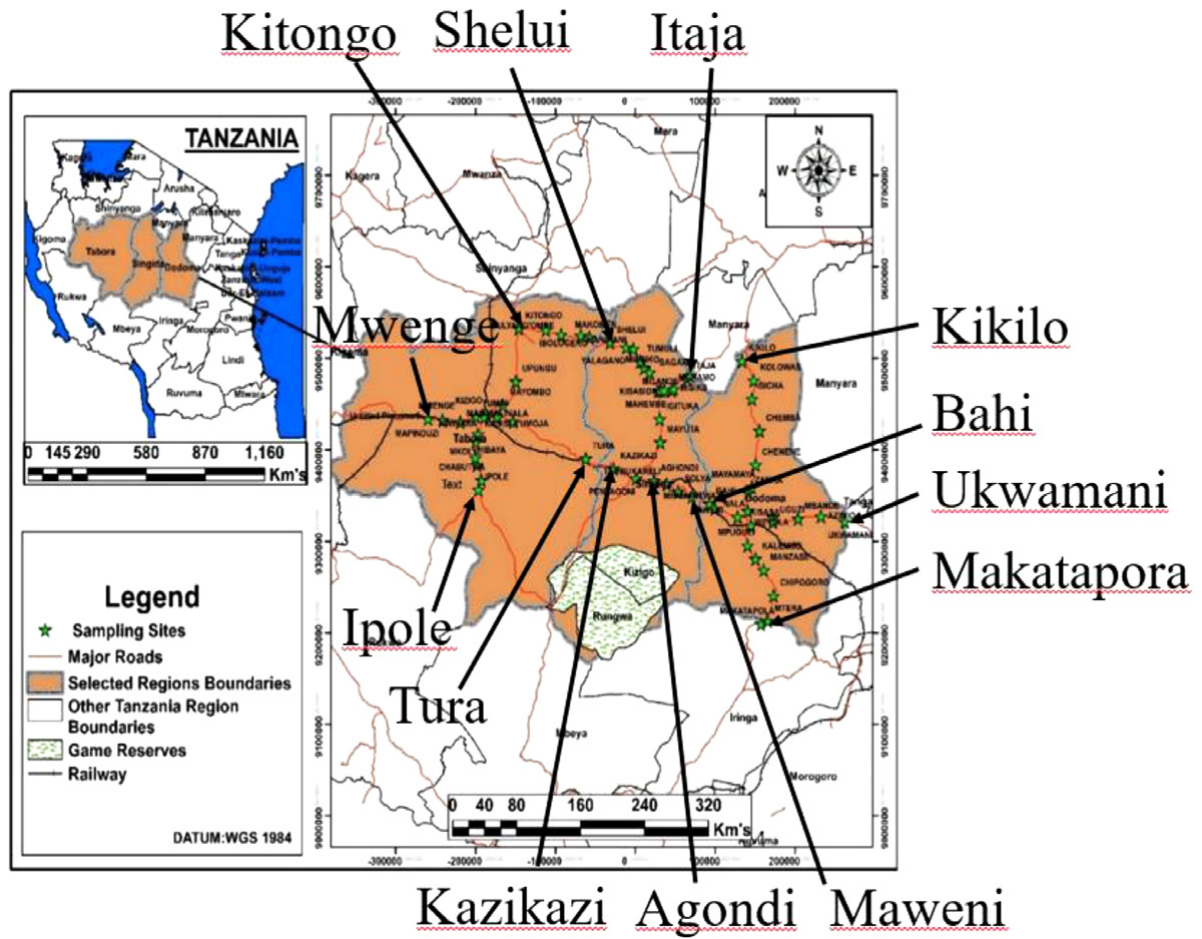
The map of sample locations in central Tanzania.

Central Tanzania is made up of three regions, which are Dodoma, Singida, and Tabora (Fig. 7). This region has major roads that cross it. In east-west directions, the major roads are B129, B6, and B143, and in north-south directions, the major roads are A104, B141, and B6. The map that shows these major roads and sample location sites that border each region is shown in Fig. 8. According to 2012 population and housing census these regions make up 12.8% of the population of Tanzania (44.9 million) and have annual growth rates of 2.1%, 2.3%, and 2.9%, respectively. The population of Dodoma, Singida, and Tabora were reported to be 2083588, 1370637, and 2291623. The region is mostly a plateau, 1100 m above sea level, and its geolocation is between the Eastern and Western Rift Valleys and within the Tanzania craton [11], [12], [13]. This region is tectonically active and contains fractured crystalline basement rocks [8]. Rocks that are found in this region include granodiorite, basalt, metavolcanic, granitoids, and granitic gneisses [14]. In the Dodoma region, where data are available, Ca^2+^+Mg^2+^was reported to exceed Na^+^+K^+^ and the dominant hydrochemical facies is reported to be a mixed Ca-Mg-Cl-SO_4_ [15].

### 3.2 Analytical Procedures

EC, NO_3_^−^, and F ^−^, and pH were analysed once. Ca^2+^, Mg^2+^, and HCO_3_^−^were determined using titration methods. Three titrations were done for each sample, and the average value is reported in the dataset. All volumes obtained from titrations were within the error margin of 2.0% of each other. pH and EC measurements were done using a High Range pH, Conductivity, and TDS Tester from Hanna Instruments (HI98130). Each sample was measured once for pH and once for EC. pH measurements was done after the instrument were calibrated with 4.0 ± 0.05, 7.00 ± 0.05 and 10.00 ± 0.05 buffer solutions. The instrument was washed with deionized water (DI) between calibration and sample testing in order to minimize cross contamination. Before EC measurements and the sample analysis, an instrument was calibrated by using a certified conductivity standard solution of 1413 µS/cm also from Hanna Instruments (HANNA HI7031) followed by a standard solution of KCl in the concentrations of 0.5 mM, 1.0 mM, 5.0 mM, 10 mM, 20 mM, and 30 mM. The phenol disulfonic method was used to determine the concentration of NO_3_^−^ ions [16]. NO_3_^−^concentrations of 0, 10, 20, 30, and 40 mg/l from standard solution were used to prepare the standard curve. Both standards and samples were taken at 410 nm. Measurements were done using a Cary 60 Uv-vis spectrophotometer from Agilent Technologies. In a conical flask, 10 mL of water sample was placed, and 25 mL of nitrate-extracting solution was added and shaken for 10 min. It was then allowed to settle for 2 min and filtered through No. 42 filter paper. A clear solution (10 mL) was pipetted into a conical flask. Then it evaporated to dryness in the oven for 24 h at a temperature of 95 °C. After dryness, the residue was cooled, and then 2 mL of phenol disulphonic acid was added rapidly, covered, and shook gently so that the reagent came into contact with the residue. After 10 min, 17 mL of cool water was added and rotated in the flask to dissolve the residue. While the flask was still cool, drops of NH_4_OH (16 mL) were added until the yellow colour was observed. After that, the volume was adjusted to 50 mL, and the solution was well mixed by gently shaking before the concentration of NO_3_^−^was measured. The SPADNS method was used to determine the concentration of F ^−^ ions.

The standard curves used to determine F ^−^ concentrations were 0.0, 0.5, 1.0, 1.5, 2.0, and 2.5 mg/l obtained from commercially available F ^−^standard solutions. Measurements were taken using a Hach DR900 handheld colorimeter (DR/890 colorimeter). A complexometric titration was used to determine TH using standard EDTA (0.01 N), 1 ml of buffer solution (1.179 g of EDTA and 0.78 g of MgSO_4_·7H_2_O), 143 mL of ammonium hydroxide, diluted to make a volume of 250 mL, and 6 drops of Eriochrome Black T (EBT). The end point was determined by the colour change from pink to blue. The volume of samples used for the calculation was the mean value of three titrations. The formula used to determine TH is CaCO_3_ mg/l.

TH as CaCO_3_ mg /l = volume of EDTA x Molarity of EDTA x 50×1000

volume of ml of sample taken for titration.

Alkalinity was determined using the formula below.

Alkalinity (HCO_3_^−^) mg/l = volume of H_2_SO_4_× Molarity of H_2_SO_4_×molar mass CaCO_3_× 1000

Volume of sample

10 mL of water sample was titrated against 0.05 M of sulphuric acid and three drops of POP indicator. Addition of POP indicator first followed by MO indicator caused the solution to remain colourless first followed by the colour to change from colourless to yellow and, when titrated with 0.05 M sulfuric acid, turned reddish pink, indicating the endpoint. Each sample was titrated three times and the mean value of sulphuric acid was used to determine HCO_3_^−^. At pH less than 8.3 which covers all samples presented in Table 1 the most predominant alkaline ion is bicarbonate (HCO_3_^−^) ions while carbonate (CO_3_^2−^) and hydroxide (OH^−^) are present at an insignificant amount.

Data processing, preparation of graphs and coefficient were done using MS Excel 2010.

## CRediT authorship contribution statement

**Jonas Didas Chondo:** Investigation, Methodology, Validation, Formal analysis, Resources. **Andrew Toyi Banyikwa:** Conceptualization, Visualization, Supervision, Project administration, Writing – original draft, Writing – review & editing.

## Declaration of Competing Interest

The authors declare that they have no known competing financial interests or personal relationships that could have appeared to influence the work reported in this paper.

## Data Availability

Dataset on the spatial distribution of groundwater quality for pH, EC, TH, Ca2+, Mg2+, HCO3- . F-, and NO3- and in Dodoma, Singida, and Tabora regions located in central Tanzania (Original data) (Mendeley Data)
